# Emerging Treatment Options for Multi-Drug-Resistant Bacterial Infections

**DOI:** 10.3390/life11060519

**Published:** 2021-06-03

**Authors:** Roberto Giurazza, Maria Civita Mazza, Roberto Andini, Pasquale Sansone, Maria Caterina Pace, Emanuele Durante-Mangoni

**Affiliations:** 1 Department of Precision Medicine, University of Campania ‘L. Vanvitelli’, Internal Medicine Section, Piazzale Ettore Ruggieri snc, 80131 Naples, Italy; roberto.giurazza@studenti.unicampania.it (R.G.); maria_civita94@virgilio.it (M.C.M.); 2 Department of Woman, Child and General & Specialized Surgery, University of Campania ‘L. Vanvitelli’, Piazza Miraglia, 80138 Naples, Italy; pasquale.sansone@unicampania.it (P.S.); mariacaterina.pace@unicampania.it (M.C.P.); 3 Unit of Infectious and Transplant Medicine, AORN Ospedali dei Colli-Monaldi Hospital, Piazzale Ettore Ruggieri snc, 80131 Naples, Italy; roberto.andini@ospedalideicolli.it

**Keywords:** antimicrobial resistance, brand-new antibiotics, old revived antibiotics, place in therapy, drug therapy, extensively drug resistant, emerging infectious diseases, resistance mechanisms

## Abstract

Antimicrobial resistance (AMR) remains one of the top public health issues of global concern. Among the most important strategies for AMR control there is the correct and appropriate use of antibiotics, including those available for the treatment of AMR pathogens. In this article, after briefly reviewing the most important and clinically relevant multi-drug-resistant bacteria and their main resistance mechanisms, we describe the emerging antimicrobial options for both MDR Gram-positive cocci and Gram-negative bacilli, including recently marketed agents, molecules just approved or under evaluation and rediscovered older antibiotics that have regained importance due to their antimicrobial spectrum. Specifically, emerging options for Gram-positive cocci we reviewed include ceftaroline, ceftobiprole, tedizolid, dalbavancin, and fosfomycin. Emerging treatment options for Gram-negative bacilli we considered comprise ceftolozane-tazobactam, ceftazidime-avibactam, meropenem-vaborbactam, imipenem-relebactam, aztreonam-avibactam, minocycline, fosfomycin, eravacycline, plazomicin, and cefiderocol. An exciting scenario is opening today with the long awaited growing availability of novel molecules for the treatment of AMR bacteria. Knowledge of mechanisms of action and resistance patterns allows physicians to increasingly drive antimicrobial treatment towards a precision medicine approach. Strict adherence to antimicrobial stewardship practices will allow us to preserve the emerging antimicrobials for our future.

## 1. Introduction

Antimicrobial resistance (AMR) remains one of the top public health issues of global concern and will remain so also in the wake of COVID-19 pandemic [1]. Among the most important strategies for AMR control there is the correct and appropriate use of antibiotics, including those available for the treatment of AMR pathogens. In this article, after briefly reviewing the most important and clinically relevant multi-drug-resistant (MDR) bacteria and their main resistance mechanisms, we describe the emerging antimicrobial options for both MDR Gram-positive cocci and Gram-negative bacilli, including recently marketed agents, molecules just approved or under evaluation and rediscovered older antibiotics that have regained importance due to their antimicrobial spectrum.

## 2. Clinically Relevant Multi-Drug-Resistant Bacteria and Their Main Resistance Mechanisms/Determinants

Resistance to multiple antibiotics currently affects both Gram-positive and Gram-negative bacteria and may theoretically involve all antimicrobial agents [2]. In response to the global phenomenon of antimicrobial resistance spread—among the most important and alarming public health issues—great efforts are being made to develop newer antimicrobial agents as well as revive older, less used molecules which have retained or regained efficacy in vitro against difficult-to-treat microorganisms [3]. A brief summary of common multi-drug-resistant/extensively drug-resistant microorganisms of clinical relevance, main resistance mechanisms, and current/emerging antimicrobial options is provided in Table 1.

When discussing ‘multi-drug-resistant’ organisms (MDRO), we currently refer to a standardized and well-accepted classification dividing clinically relevant bacteria into multi-drug resistant (MDR), extensively drug resistant (XDR), and pan-drug resistant (PDR) [4]. This classification can be applied to results of antimicrobial susceptibility assays on bacterial isolates throughout a range of clinical or experimental microbiology laboratories and allows for a common ground for clinical practice and clinical research. In this classification, MDR is defined as nonsusceptibility (or intermediate resistance) to at least one molecule in three or more categories of antibiotics; this result must obviously be obtained from in vitro antimicrobial susceptibility testing. XDR is defined as nonsusceptibility to at least one agent in all but two, or fewer than two, antibiotic classes (i.e., the isolated bacterium remains susceptible to only one or two classes of antibiotics). By contrast, PDR is defined as the lack of susceptibility to all agents in all antibiotic classes tested or available in a defined setting. It is important to underscore that when using these definitions of resistance for specific organisms or groups of microorganisms, one should not consider antimicrobial agents for which a bacterium exhibits intrinsic resistance or large-scale acquired resistance [4]. On the other hand, the emerging availability of newer antibiotics necessarily changes prior definitions and often results in the recategorization of an XDR or PDR as MDR or XDR, respectively.

Common MDROs include, among Gram-positives, methicillin-resistant *Staphylococcus aureus* (MRSA), ampicillin- or vancomycin-resistant enterococci (ARE/VRE), and penicillin-resistant *Streptococcus pneumoniae* (PRP) [5]. Difficult-to-treat Gram-negative bacteria include extended-spectrum β-lactamase (ESBLs) producing bacilli and carbapenem-resistant Gram-negatives (mostly Enterobacterales, *Pseudomonas* spp., *Acinetobacter baumannii*). Most commonly encountered MDR/XDR Gram-negative rods include *Enterobacter* spp., *Escherichia coli*, *Klebsiella pneumoniae*, *A. baumannii*, and *Pseudomonas aeruginosa*. Less common but still clinically relevant are *Stenotrophomonas malthophilia*, *Burkolderia cepacian*, and *Achromobacter xylosoxidans* [6,7]. These three bacteria are emerging nosocomial pathogens, associated with opportunistic infections in severely immunocompromised patients, such as those affected by cystic fibrosis, cancer, HIV, or neutropenia, and in ICU patients, where mechanical ventilation, central venous catheters, or other types of indwelling catheters and broad-spectrum antibiotic use are common. These germs have the ability to colonize, creating biofilm and humid surfaces, including medical devices, and exhibit an intrinsic resistance to most broad-spectrum antibiotics, including carbapenems, aminoglycosids and polymixin B [8,9,10,11].

The majority of the aforementioned resistant Gram-positive and Gram-negative bacteria was defined as part of the ‘ESKAPE’ group (*Enterococcus faecium*, *Staphylococcus aureus*, *Klebsiella pneumoniae*, *Acinetobacter baumannii*, *Pseudomonas aeruginosa*, and *Enterobacter* spp.) [12].

The most successful resistant Gram-positive coccus has been *S. aureus*. It can develop resistance to almost all molecules used to treat Gram-positive bacteria, although to a variable extent [13]. *S. aureus* is often resistant to penicillin, due to the production of β-lactamases, and methicillin, due to the mutation of transpeptidase binding site of PBP2 (giving rise to PBP2a). It can become nonsusceptible to vancomycin as vancomycin intermediate strains (VISA), due to multiple mutations leading to a thickened, poorly crosslinked cell wall, or true vancomycin resistant strains (VRSA) harboring *vanA*- or *vanB*-mediated replacement of the D-Ala-D-Ala binding site by D-Ala-D-Lactate within the peptidoglycan [14]. More recently, *S. aureus* was found to potentially develop daptomycin resistance due to alteration in the cell membrane composition, changing its electrical charge and thus its binding propensity to the positively charged lipopeptide molecule, as well as to a thickened cell wall (as commonly seen in VISA strains) [15]. Beyond its resistance to cell wall active agents, *S. aureus* may be characterized by variable degrees of resistance to multiple other antimicrobial classes, including clindamycin (methylation of the ribosomal binding site), linezolid (mutations at the 23S rRNA ribosomal binding site or *cfr* efflux pump expression, rifampicin (mutations at one of several sites at the RNA polymerase binding site), or doxycycline (efflux pump or ribosomal protection proteins expression). In *S. aureus*, the presence of methicillin-resistance implies MDR, regardless of resistance to other antibiotic classes [16].

In enterococci, resistance mostly affects three drug classes: penicillin/ampicillin, high-level gentamicin, and vancomycin. Penicillin/ampicillin resistance mostly stems from decreased binding affinity at the transpeptidase binding sites or overexpression of PBP5, much less commonly from β-lactamases expression, and is therefore largely unaffected by β-lactamase inhibitor treatment. Vancomycin-resistance, more common in *E. feacium* strains, originates from the replacement of the D-Ala-D-Ala binding site by either D-Ala-D-Lactate (due to the *vanA* or *vanB* operon) or D-Ala-D-Serine (due to *vanC*, *vanE*, or *vanG* operons). Whilst intrinsic resistance to gentamicin derives from decreased cell wall permeability or low-level expression of aminoglycoside-modifying enzymes, high-level resistance to gentamycin is common and results from high-level expression of aminoglycoside-modifying enzymes [17]. These MDR/XDR enterococci often retain susceptibility to linezolid, which, however, can also be attenuated by mutations at the 23S rRNA ribosomal binding site or by expression of *cfr* plasmid-mediated methylases [18].

MDR streptococci are also emerging, although not to the extent of other pathogenic Gram-positive cocci. Among *S. pneumoniae*, there are >90 capsular serotypes, differing not only in virulence but also in prevalence and extent of antimicrobial drug resistance. Serotypes most frequently involved in pneumococcal disease or colonization are also often MDR. The most common mechanisms of resistance include PBP alterations due to transferable genetic elements/plasmids affecting penicillin, ampicillin, and amoxicillin (I or R); ribosomal methylases, such as *ermB*, encoded by plasmids and conferring high-level resistance to macrolides and lincosamides; efflux pump acquisition (e.g., *mef*) transferred via plasmids and causing high-level resistance to macrolides and fluoroquinolones; and gyrase/topoisomerase IV chromosomal mutations impairing susceptibility to levofloxacin and moxifloxacin. As a mere example, in Italy, the current prevalence of resistance in *S. pneumoniae* is 10–15% for penicillin, 6–11% for ceftriaxone, and 25–35% for macrolides. Penicillin- and cephalosporin-resistance is also emerging among viridans-group streptococci, which are also able to rapidly develop on treatment in vivo resistance to daptomycin [19].

The major mechanism of resistance of Gram-negative bacilli to β-lactams is the production of β-lactamases (Blac), enzymes that hydrolyze the β-lactam ring [20]. The Ambler classification of Blac groups these enzymes into four major families (A-D) [21]. Ambler class A Blac includes enzymes present in Enterobacterales, such as TEM and SHV—that can be inactivated by clavulanate, sulbactam, and tazobactam-extended spectrum β-lactamases (ESBL) and *K. pneumoniae* carbapenemases (KPC and GES) [22]. Carbapenem-resistant Gram-negatives (including not only carbapenemase-producing Enterobacterales (CRE) but also *Pseudomonas* spp. and *Acinetobacter* spp.) are current ‘nightmare bacteria’ according to the US Centers for Disease Control and Prevention and one of the greatest ongoing threats for human health. These microorganisms are increasingly recognized as causes of disease, both in acute hospitals and long-term care facilities. A typical feature of CREs and other carbapenem-resistant bacilli is their association with poor clinical outcomes, with short-term lethality rates of 40–50%. Class B Blac includes the metallo-β-lactamases (MBLs), such as New Delhi MBL (NDM), Verona integrin-encoded MBL (VIM), and imipenemases (IMP). At present, these Blac can be fought by a very limited number of antimicrobial agents. Class C includes the AmpC Blac group, which can be encoded by either chromosomal or plasmid genes and can therefore confer a variable degree of resistance to antimicrobial agents. Ambler Class D Blac mostly includes the oxacillinases (OXA), such as OXA-48, which has a broad spectrum of inhibition, including carbapenems, and is a common resistance mechanism among Enterobacterales, and OXA-23 and OXA-51-like enzymes, which are mostly found in *A. baumannii* [21]. Precise knowledge of resistance mechanisms in place in each MDR/XDR pathogen is needed to understand limitations of current molecules and place in therapy of emerging options. For instance, avibactam, a newer generation β-lactamase inhibitor, binds and inhibits both KPC, class C and OXA-48, but not MBLs. Vaborbactam and relebactam efficiently inhibit only KPC and class C, but not OXA-48, and are inactive against MBLs [23].

**Figure 1:**
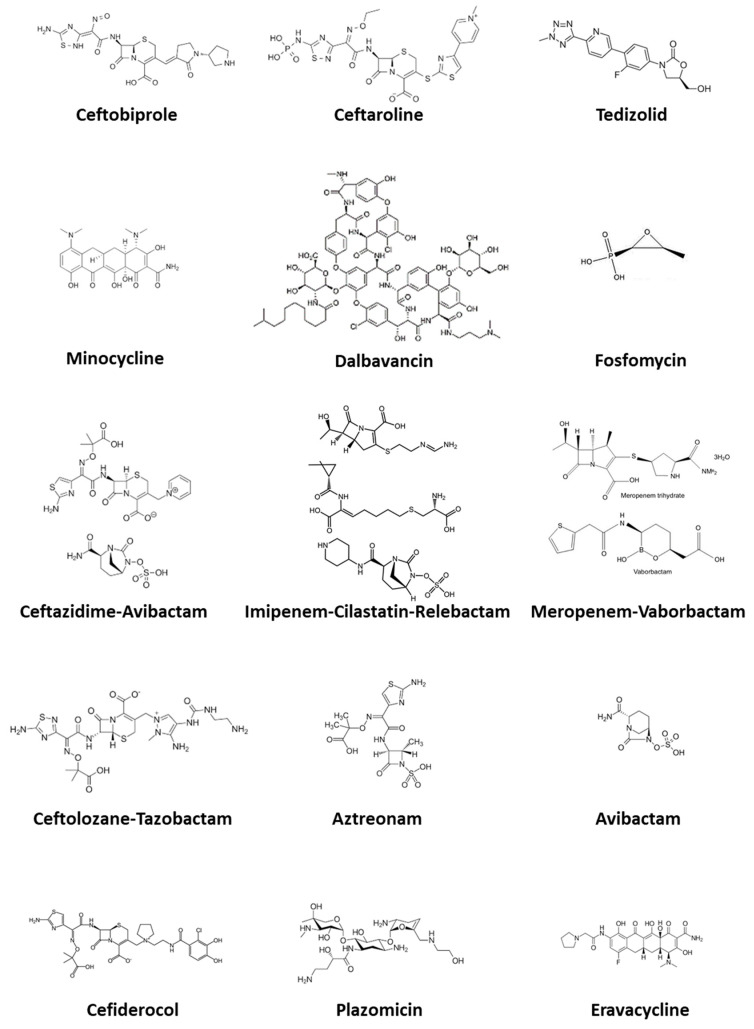
Chemical structure of the antimicrobial agents discussed in this article.

As summarized above, knowledge of AMR mechanisms together with the development of newer agents is translating into a challenging and exciting scenario where precision medicine applies to bacterial infection treatment. In this review, we highlight the most important features of emerging antibiotics for MDR infections, addressing both newer agents as well as old, less used, and recently revived molecules. In Figure 1 we show the chemical structure of the molecules considered.

Our review is based on a comprehensive literature search of PubMed, Embase, Scopus, and Google Scholar, from inception to 31 March 2021.

## 3. Emerging Antimicrobial Options for MDR Gram-Positive Cocci

### 3.1. Ceftobiprole

Ceftobiprole medocaril is a fifth-generation cephalosporin approved for the treatment of hospital-acquired bacterial pneumonia (HABP), excluding ventilator-associated bacterial pneumonia (VABP), and community-acquired pneumonia (CAP). Ceftobiprole exerts its antibacterial activity by binding to important penicillin-binding proteins and inhibiting their transpeptidase activity, which is essential for the synthesis of bacterial cell walls. These include PBP2a, making ceftobiprole the only β-lactam (together with ceftaroline) active against MRSA. It is rapidly converted to the active metabolite ceftobiprole following intravenous administration [24].

Ceftobiprole has a broad spectrum of activity, notably including methicillin-resistant *S. aureus* and coagulase-negative Staphylococci, penicillin-resistant *S. pneumoniae*, and, although to a lesser extent, *E. faecalis*. Similar to cefepime, ceftobiprole is also active against some MDR Gram-negative bacilli, including AmpC-producing *E. Coli* and *P. aeruginosa*, but not ESBL-producing strains [25,26].

Ceftobiprole is primarily excreted renally by glomerular filtration, with minimal propensity for interaction with coadministered drugs. The recommended dose is 500 mg, administered by 2 h intravenous infusion every 8 h, with dose adjustments according to renal function. Of note, little diffusion of this molecule to the gut lumen has been observed, possibly accounting for its low propensity to select for *Clostridioides difficile* [27].

In a phase III trial in patients with HABP, ceftobiprole monotherapy was as efficacious as the combination of ceftazidime and linezolid in terms of both clinical and microbiological cure and was noninferior to ceftazidime/linezolid in the subgroup of patients with HABP, but excluding VABP. Ceftobiprole and ceftazidime/linezolid were similarly well tolerated. Based on current evidence, Ceftobiprole is an efficacious and well-tolerated option for empirical treatment of patients with HABP (excluding VABP) [28].

Campanile et al. investigated the in vitro susceptibility of ceftobiprole and its potential synergistic activity in combination with other antimicrobials against 46 selected Gram-positive pathogens displaying resistance or decrease susceptibility to several drugs. The gradient-cross method was used to assess synergism between ceftobiprole and daptomycin, levofloxacin, linezolid, rifampicin, and piperacillin/tazobactam. Ceftobiprole plus daptomycin was synergistic against all isolates. Ceftobiprole plus linezolid was synergistic against four isolates belonging to different species (MRSA/VSSA, *S. epidermidis*, *E. faecium*, and *E. faecalis*). Ceftobiprole plus levofloxacin was synergistic only against enterococci. In conclusion, ceftobiprole exhibited a potent in vitro antibacterial activity and good synergy with daptomycin against a range of tested Gram-positive isolates, despite their antibiotic resistance phenotypes. The use of ceftobiprole alone or in combination may therefore provide a promising alternative therapy for the treatment of infections caused by resistant Gram-positive bacteria [29].

### 3.2. Ceftaroline

Ceftaroline is another fifth-generation cephalosporin approved for the treatment of CAP and acute bacterial skin and skin structure infections (ABSSSIs). It recently received an additional approval for the treatment of *S. aureus* bacteremia (SAB) associated with ABSSSIs. Ceftaroline has shown efficacy for the treatment of methicillin-resistant SAB, including for isolates with elevated minimum inhibitory concentrations to conventional therapy, when used alone or in combination with other agents. In multiple studies, ceftaroline displayed rapid bloodstream eradication, even in the setting of refractory MRSA SAB or infective endocarditis [26,30].

It has activity against MDR Gram-positive bacteria, including MRSA, VRSA (vancomycin-resistant *S. aureus*), and respiratory pathogens such as *S. pneumoniae* (including multi-drug-resistant strains), *Haemophilus influenzae*, and *Moraxella catarrhalis*. Mirroring other broad-spectrum cephalosporins, ceftaroline does not possess activity against extensively resistant Gram-negative bacilli and exhibits limited activity against most nonfermentative Gram-negative bacilli (e.g., *P. aeruginosa, Acinetobacter* spp.) as well as many anaerobic species [31].

The recommended duration of treatment is 5–14 days for cSSTI and 5–7 days for CAP, and the standard dose in adults with normal renal function is 600 mg by 1 h intravenous infusion every 12 h. A higher dose of 600 mg every 8 h can also be used in more severe cases or in SAB. In adults with a creatinine clearance <50 mL/min, ceftaroline dose should be reduced. If the creatinine clearance is between 30 and 50, the recommended dose is 400 mg every 12 h; if the creatinine clearance is between 15 and 30 mL/min, the recommended dose is 300 mg every 12 h; and if creatinine clearance is less than 15 mL/min, the recommended dose is 200 mg every 12 h [32].

The most common adverse reactions reported in ≥3% of approximately 3242 patients treated in clinical trials were diarrhea, headache, nausea, pruritus, and were generally mild or moderate in severity. Diseases associated with *C. difficile* (diarrhea) can be observed. In addition, ceftaroline lowers the epileptogenic threshold [33].

In phase 3, multicenter, randomized, and double-blind studies, Corey at al. evaluated the safety and efficacy of ceftaroline in a comparative fashion. Noninferiority was observed, and satisfactory clinical cure rates were achieved by ceftaroline (600 mg every 12 h) compared to vancomycin plus aztreonam (1 g each every 12 h) for 5–14 days in complicated skin and skin-structure infections [34].

### 3.3. Dalbavancin

Dalbavancin is a lipoglycopeptide approved in US and Europe for the treatment of ABSSIs caused by Gram-positive bacteria. Like other members of its family (telavancin and oritavancin, not yet widely approved), dalbavancin is an analogue of glycopeptides incorporating structural modifications responsible for novel and somehow improved pharmacokinetic and pharmacodynamic features [26,35]. Lipoglycopeptides act by blocking cell wall synthesis and binding to the D-Ala-D-Ala terminus of the pentapeptide peptidoglycan precursors; in addition, they anchor to the cell wall with high affinity thanks to their lipophilic lateral chain. Moreover, the addition of a lateral lipophilic chain gives dalbavancin a unique pharmacokinetic profile, with a very long half-life (>10 days) [36].

Dalbavancin has in vitro activity against sensible and MDR Gram-positive bacteria, including MRSA, methicillin-resistant coagulase negative staphylococci and VRE, with the exception of those with a vanA phenotype.

It can be administered in two ways: either as a single IV dose of 1500 mg over 30 min, or as a two-dose regimen, with an initial 1000 mg IV dose over 30 min, followed by 500 mg one week later. Dose adjustments are required only for patients with severe renal dysfunction (CrCl < 30 mL/min) [36]. It shows a high bone penetration and a favorable safety profile when administered weekly up to 8 weeks.

The unique pharmacokinetic profile of dalbavancin allows treatment of MDR infections with a once or twice weekly administration, thus avoiding hospitalization or decreasing length of hospital stay and overall general costs and decreasing all the risks connected to long-term indwelling venous catheters that are needed for other daily administered antibiotics (such as daptomycin). Dalbavancin appears specifically ideal for treatment in an outpatient setting. Bouza et al. reported an overall clinical success rate of 84.1% with dalbavancin when treating ABSSIs, osteomyelitis, prosthetic joint infections, and catheter-related bacteremia, with an average cost reduction of 3064 € per patient [37].

In a recent meta-analysis including 7 RCTs and 2665 patients, Y. Wang et al. showed that dalbavancin was comparable to other antibiotics in treating chronic Gram-positive infections in terms of efficacy and safety, and that the dual-dose regimen showed a better safety profile compared with the single-dose regimen in the treatment of ABSSSIs. Specifically, clinical response to dalbavancin was better in catheter-related bloodstream infections (CRBSIs) and osteomyelitis, but no significant difference was observed in terms of adverse events between dalbavancin and other treatments [38].

### 3.4. Tedizolid

Tedizolid phosphate is a newer oxazolidinone prodrug that is rapidly converted in its microbiologically active counterpart, tedizolid, by endogenous phosphatases [39]. Similar to linezolid, it inhibits bacterial protein synthesis binding to 23S-rRNA of the bacterial ribosomal subunit 50S. Moreover, tedizolid overcomes the most important mechanism of resistance to linezolid, which is the methylation of the 23S rRNA subunit by the enzyme *cfr* [40].

Tedizolid is mainly active against Gram-positive bacteria, such as *Enterococcus* spp. (including vancomycin-resistant strains), *Staphylococcus* spp. (including methicillin-resistant strains), and *Streptococcus* spp. Its in vitro activity against these bacteria is 4–8 times greater than linezolid, meaning that it can be used at lower doses [41]. Tedizolid is indicated in the treatment of ABSSSIs caused by Gram-positive bacteria, even if resistant to glycopeptides, daptomycin, and linezolid. It can be administered orally or intravenously at the same dosage (200 mg once daily), since its oral bioavailability is almost complete. The pharmacodynamic parameter that best describes tedizolid’s efficacy is AUC/MIC [42].

In the randomized, double-blind, phase 3, noninferiority ESTABLISH-2 trial, tedizolid (200 mg once daily for 6 days) was found to be noninferior in efficacy to linezolid (600 mg twice-daily for 10 days) for the treatment of ABSSSIs and to be similarly tolerated, but with a lower incidence of gastrointestinal AEs and bone marrow suppression than linezolid [43].

### 3.5. Fosfomycin Disodium

Fosfomycin disodium is a recently redeveloped formulation of an old cell wall active agent. It has a very simple structure and a low molecular weight, favoring its penetration into the bacterial cell wall. Entry is achieved through transport systems utilized by alpha glycerol-phosphate and glucose-6-phosphate, key elements of the bacterial metabolism; this explains the often reduced fitness and virulence of fosfomycin-resistant bacterial strains [44]. Fosfomycin inhibits an early step of the peptidoglycan synthesis, blocking formation of the N-acetylmuramic acid, thus acting in direct synergism with β-lactams. Fosfomycin is active in the log-phase of bacterial growth and exerts bactericidal effects against a broad spectrum of pathogenic bacteria, including Gram-positive (e.g., *S. aureus* and *E. faecalis*) and Gram-negative bacteria (e.g., *E. coli* and *Klebsiella* spp.). Importantly, fosfomycin activity has been documented against approximately 80% of CRE, especially KPC-producing *K. pneumoniae*, including a proportion of colistin-resistant strains. As a result of its mechanism of action and renal safety profile, fosfomycin is a good option to exploit synergism in combination with aminoglycosides [45].

Fosfomycin disodium reaches high serum concentrations when dosed at 4 g every 6 h intravenously. It retains adequate penetration into various tissues, including lung, central nervous system, and bone [46]. However, when used as monotherapy, rapid onset of resistance to fosfomycin is observed. The resistance mechanisms include reduced intracellular transport of the antibiotic changes in targets and direct inactivation by metalloenzymes and kinases [47]. These features strongly support the need to always combine fosfomycin with other antimicrobials, including β-lactams, aminoglycosides, daptomycin, and newer molecules such as ceftaroline, ceftobiprole, and ceftazidime-avibactam. Fosfomycin is a well-tolerated drug, being its most common adverse reactions nausea, vomiting and diarrhea, and skin rashes. However, long courses of high-dose fosfomycin disodium are associated with significant sodium and water retention, which may be difficult to manage in patients with concurrent decompensated heart failure, renal failure, or liver cirrhosis [44]. Electrolyte disturbances are also common during high-dose fosfomycin administration.

## 4. Emerging Antimicrobial Options for MDR Gram-Negative Rods

### 4.1. Ceftolozane-Tazobactam

Ceftolozane-tazobactam is a combination of a novel cephalosporin with an established β-lactam β-lactamase inhibitor, approved for the treatment of cIAI (in combination with metronidazole) and cUTI, including pyelonephritis [48].

It has a similar spectrum of antimicrobial activity, compared to ceftazidime-avibactam, with some important differences. Ceftolozane is notable for its potent activity against *P. aeruginosa*, with high affinity to PBP1b, PBP1c, and PBP3, as well as for its stability to AMPc β-lactamases. Its activity is less affected by efflux pump expression or by deletion of the outer membrane protein OprD in *P. aeruginosa*. Tazobactam is an established β-lactam β-lactamase inhibitor that can inhibit only some Ambler class A β-lactamases (TEM, SHV, CTX-M), but not KPC, nor Class B (metallo-β-lactamases), nor class D (OXA-48) and class C only in high concentrations. It is therefore active against Gram-negative bacteria, including MDR *P. aeruginosa* and ESBL-producing Enterobacteriaceae, but *Acinetobacter* spp. and *Stenotrophomonas* spp. are generally resistant [49,50].

Ceftolozane-tazobactam is available for intravenous use only, with a currently approved dosage of 1 g of ceftolozane and 500 mg of tazobactam every 8 h, for adult patients with an estimated creatinine clearance of >50 mL/min. More recently, a 3 g every 8 h dosing regimen has been approved for HABP. It has a relatively short mean plasma half-life of 2.7 h in healthy adults, and this accounts for the need to administer a dose every 8 h [51]. Both ceftolozane and tazobactam are cleared through the kidneys and the clearance of tazobactam does not appear to be influenced by coadministration of ceftolozane (in contrast to piperacillin).

In the phase 3, randomized, double-blind, double-dummy, noninferiority ASPECT-cUTI trial ceftolozane-tazobactam was compared to levofloxacin in the treatment of cUTIs and was found to be superior to the latter in clinical cure and microbiological eradication both in the intention-to-treat and in the per protocol analysis. Instead, outcomes were similar when only patients with baseline levofloxacin-susceptible pathogens were analyzed [52,53].

In another phase 3, randomized, double-blind trial (ASPECT-cIAI trial), Solomkin et al. demonstrated that ceftolozane-tazobactam plus metronidazole was noninferior to meropenem in the treatment of patients with cIAIs, with similar frequency of adverse events [54]. Of note, in patients with moderate renal failure a lower cure rate was observed in the ceftolozane-tazobactam plus metronidazole arm. This finding prompted the FDA to include a warning to monitor renal function at least daily and to change the dosing accordingly [52].

### 4.2. Ceftazidime-Avibactam

Ceftazidime-avibactam is a β-lactam/β-lactamase inhibitor, approved for the treatment of complicated urinary tract infections (cUTI), complicated intraabdominal inflections (cIAI) in combination with metronidazole, and HABP, including VABP, when no other treatment options are available [55]. It is a combination of a known third generation cephalosporin with broad spectrum activity against Gram-negative bacilli (including *P. aeruginosa*) and a non-β-lactam β-lactamase inhibitor, which is active against class A (such a ESBLs, *K. pneumoniae* carbapenemases), class C (AmpC), and some class D (OXA-48) β-lactamases. However, it is not active against MBLs (such as New Delhi metallo-β-lactamases) [56,57].

Avibactam expands ceftazidime’s spectrum of activity to include highly resistant Gram-negative pathogens, including many AmpC-, ESBL-, and KPC carbapenemase-producing strains. It is important to underscore that most strains express at the same time multiple Blac, hence the major role of avibactam in fighting these infections. Ceftazidime-avibactam is active against most Enterobacterales and *P. aeruginosa*, but not XDR *A. baumannii*, which is largely resistant to ceftazidime-avibactam; it has a limited activity against anaerobic bacteria (hence, the combination with metronidazole in cIAIs) and Gram-positive bacteria. Compared to other β-lactamase inhibitors, the advantages of avibactam are its long half-life, small molecular size and molecular weight, polarity, interaction with important amino acids near the active catalytic sites of β-lactamases, reversibility of inhibition, and the low potential for resistance induction [58,59].

The pharmacokinetics of ceftazidime-avibactam is similar to that of β-lactam molecules. It is a hydrophilic drug; therefore, its volume of distribution is mostly restricted to the extracellular volume. Its penetration in the lung tissue and epithelial lining fluid is low, around 30% of plasma concentrations, but its activity is not influenced by the surfactant. It does not interact with membrane transport proteins nor with cytochrome P450 enzymes [58].

As for other cephalosporins, the best predictor of its antimicrobial activity is the proportion of the dosing interval in which the drug concentration is in excess of the MIC (%T/MIC). Ceftazidime-avibactam, as other β lactams, primarily exerts a bactericidal effect through binding of PBP and inhibition of cell wall synthesis [60].

The most common mechanism of resistance against ceftazidime-avibactam is the expression of efflux pumps or a mutation in the omega-3 domain of the KPC β-lactamase, which makes it refractory to inactivation by avibactam; often, this mutation is associated with restoration of susceptibility to meropenem. Ceftazidime-avibactam becomes inactive when other Blac, most class B and class D enzymes, are expressed [61].

The currently approved doses for adult patients with an estimated creatinine clearance >50 mL/min are ceftazidime 2000 mg + avibactam 500 mg every 8 h, given as an intravenous infusion of at least 2 h [58]. Both ceftazidime and avibactam are primarily cleared through the kidneys; therefore, their dosages must be adjusted according to renal function. Both are eliminated by renal replacement therapies and the dose should be given after hemodialysis in patients with end-stage renal disease. The mean plasma half-life of ceftazidime is 1.5 h [58].

Adverse reactions are uncommon and may include candidiasis, *C. difficile* diarrhea, rash, urticaria, infusion-site reactions, thrombocytosis, eosinophilia, and direct Coombs test positivity.

In 2018, van Duin et al. showed that ceftazidime-avibactam may be a reasonable alternative to colistin in the treatment of *K. pneumoniae* carbapenemase-producing CRE infections. In patients treated with ceftazidime-avibactam versus colistin, all-cause hospital mortality 30 days after starting treatment was 9% versus 32%, respectively. Moreover, at 30 days, patients treated with ceftazidime-avibactam, compared with those treated within colistin, had a 64% probability of a better outcome [62]. Real-world data have subsequently confirmed the superiority of ceftazidime-avibactam to polymyxin-based therapies.

The lack of in vitro activity of ceftazidime-avibactam against MBL and the fact that many MBL producers also coproduce other β-lactamases (such as ESBLs, AmpC, OXA-48, etc.) have led us to hypothesize a potential effect of combining ceftazidime-avibactam with aztreonam, which is not hydrolyzed by MBLs per se. Synergistic effects have been seen in in vitro and in vivo studies. This raises the possibility of using atypical combinations of BLBLIs and other β-lactams, such as piperacillin-tazobactam plus aztreonam and ceftazidime-avibactam plus aztreonam, against MBL and ESBL producers [63]. Ceftazidime-avibactam may be considered the cornerstone in the treatment of severe infections due to KPC- and OXA-48-producing Enterobacteriaceae, due to avibactam’s unique inhibitory profile against OXA-48 and ceftazidime’s stability to hydrolysis by this enzyme.

### 4.3. Meropenem-Vaborbactam

Meropenem-vaborbactam is the combination of a well-known carbapenem with a new β-lactamase inhibitor, which was FDA approved in August 2017 for the treatment of adults with cUTI, including pyelonephritis.

Vaborbactam is a first-in-class, cyclic boronic acid β-lactamase inhibitor with activity against Ambler class A and C enzymes, including KPC, with limited activity against class D oxacillinases, and no activity against MBL. Combined with meropenem, it restores antimicrobial effects against KPC-producing CRE [50]. Vaborbactam has shown a pharmacokinetic profile similar to that of meropenem. The pharmacokinetic/pharmacodynamic parameters that correlate with efficacy include time above the minimum inhibitory concentration (MIC) for meropenem and overall exposure (measured by the area under the concentration-time curve [AUC]) for vaborbactam [64].

The approved regimen is 2 g of meropenem and 2 g of vaborbactam q8h with a 3 h infusion time for the treatment of infections caused by KPC-positive CRE isolates with meropenem MICs as high as 8 mg/L [65].

The targeting antibiotic nonsusceptible Gram-negative organisms (TANGO)-I trial evaluated safety and effectiveness of Meropenem-vaborbactam compared to piperacillin-tazobactam in cUTI. The authors concluded that among patients with cUTI, Meropenem-vaborbactam was noninferior to piperacillin-tazobactam in complete resolution or improvement of symptoms along with microbial eradication [66].

The TANGO II, a phase 3 randomized trial, investigated efficacy and safety of Meropenem-vaborbactam versus best available therapy (BAT) in adults with serious infections due to CRE. It showed that Meropenem-vaborbactam was associated with higher rates of clinical cure than BAT at both end of treatment (difference 32.3%; 95% CI 3.3–61.3%, *p* = 0.032) and test of cure (difference 32.7%, 95% CI 4.6–60.8%, *p* = 0.02) and with a reduced all-cause mortality. Moreover, it was associated with fewer adverse events than BAT, including lower incidence of nephrotoxicity [67].

### 4.4. Imipenem-Cilastatin-Relebactam

Imipenem-cilastatin-relebactam is yet another combination of a new β-lactamase inhibitor with a well-known carbapenem along with its partner cilastatin, which is a dehydropeptidase inhibitor that improves in vitro stability of imipenem. It has been recently approved for the treatment of complicated urinary tract infections (cUTIs) and complicated intra-abdominal infections (cIAIs) [68].

Relebactam is a non- β-lactam β-lactamase inhibitor, chemically similar to avibactam and with a similar inhibitory mechanism. It has activity against Ambler class A and class C carbapenemases in vitro, but not against metallo-β-lactamases (Ambler class B) nor OXA-48 (Ambler class D). However, its activity against non-fermenting Gram-negative bacteria (such as *A. baumannii* and *S. maltophila*) is limited [68,69].

In patients with normal creatinine clearance, the standard dose regimen is imipenem 500 mg cilastatin 500 mg relebactam 250 mg, administered via 30 min intravenous infusion every 6 h. In patients with renal dysfunction, the dose should be reduced, but the duration of the infusion and the dosing interval should remain the same.

A phase 2, multicenter, double-blind, comparative clinical trial evaluated safety, tolerability, and efficacy of relebactam at two doses plus imipenem in the treatment of patients with cIAI. A dose of 500 mg of imipenem plus 250 mg of relebactam (with dose adjustments for patients with decreased renal function), administered intravenously q6h as a 30 min infusion) allows one to achieve, in the majority of patients, exposures that lie within the therapeutic window [70].

Both imipenem and relebactam have good lung penetration, indicating a potential efficacy in VABP. From a pharmacodynamic standpoint, the best marker of imipenem-relebactam efficacy is imipenem time above dynamic imipenem MIC (t/MIC) [71], although in another study Bhagunde and colleagues described the ratio of AUC for free drug to MIC (AUC/MIC) as the primary indicator of efficacy [72].

The phase 3 multicenter, randomized, double-blind RESTORE-IMI 1 trial compared imipenem-relebactam to imipenem+colistin in the treatment of HABP/VABP, cIAI, or cUTI caused by imipenem-nonsusceptible bacteria. Favorable overall response was similar among the treatment groups, but it was higher in patients with *P. aeruginosa* treated with imipenem-relebactam. Favorable clinical response at day 28 was higher among those treated with imipenem-relebactam, as was 28 day all-cause mortality. Serious adverse events (AEs), as well as nephrotoxicity, occurred more often in patients treated with imipenem-colistin [73].

The phase 3 multicenter, randomized, double blind RESTORE IMI 2 trial compared imipenem-relebactam to piperacillin-tazobactam in the treatment of HABP and VABP. Imipenem-relebactam was noninferior (*p* < 0.001) to piperacillin-tazobactam for both endpoints: day 28 all-cause mortality and favorable clinical response at early follow-up. Serious adverse events (AEs) occurred similarly in the two groups, and no fatal adverse events were reported [74].

### 4.5. Plazomicin

Plazomicin is a new aminoglycoside, derived from sisomicin, that inhibits protein synthesis by binding to ribosomal 30S subunit of bacteria. It has a broad-spectrum in vitro activity against *K. pneumoniae* and other Gram-negative bacteria, including carbapenem-resistant strains. It has been designed to resist the most common aminoglycoside-modifying enzymes (AME), which are often expressed by carbapenem-resistant Enterobacterales [75]. Being an aminoglycoside, its mechanism of action is clearly independent of the carbapenem-resistance mechanism (unlike the new β-lactam/β-lactamase inhibitors). It is therefore active against KPC-producers as well as CREs with mechanisms other than NDM. Indeed, CRE specifically producing NDM are most likely to coexpress 16S rRNA methylases, that inactivate all aminoglycosides, including plazomicin [76].

Plazomicin has been approved for cUTI and acute pyelonephritis, based on the results of the EPIC study, a phase 3 noninferiority RCT comparing plazomicin to meropenem, both followed by oral levofloxacin where needed [77]. Its recommended dose regimen is 15 mg/kg/q24h in patients with a normal renal function. Dose reductions and therapeutic drug monitoring are warranted in patients with moderate or severe renal impairment [78].

The multicenter, randomized, open-label CARE trial evaluated the efficacy and safety of plazomicin compared to colistin as part of a combination therapy for serious CRE bloodstream infections, HABP or VABP, but was stopped prematurely because of slow enrollment. In patients treated with plazomicin, data showed a lower percentage of death from any cause at day 28 or clinically significant disease-related complications among those with BSI but higher in those with HABP/VABP. In a secondary analysis of time to death, numerically fewer deaths were reported at day 14 and at day 60 among patients who received plazomicin-based regimens [78].

The most common adverse reactions associated with plazomicin are decreased renal function, diarrhea, hypertension, headache, nausea, vomiting, and hypotension. As with other aminoglycosides, plazomicin may cause neuromuscular blockade, ototoxicity, and fetal harm in pregnant women [79].

An interesting feature of aminoglycosides is their potential synergistic activity with β-lactams, as a result of both increased permeability when the bacteria are exposed to β-lactams (which are cell wall synthesis inhibitors) and reduction in carbapenemase-production caused by the aminoglycosides (which are protein synthesis inhibitors) [80].

### 4.6. Minocycline

Minocycline is a very old semisynthetic tetracycline derivative, first approved by FDA in the 1960s, which has recently regained attention for its potential effectiveness in MDR Gram-negative VABP. It is a broad-spectrum antibiotic with activity against both aerobic and anaerobic Gram-positive and Gram-negative bacteria. It acts by inhibiting protein synthesis, binding to bacterial ribosomal 30S subunit; thus, it is a bacteriostatic agent. In vitro and in vivo studies recently established the utility of minocycline against MDR *Acinetobacter* spp. [81].

Minocycline is available for intravenous and oral use and, when administered orally, it is readily absorbed by the gastrointestinal tract with an optimal bioavailability, allowing a switch from IV to oral formulation without loss of efficacy. In adults, the recommended initial dose is 200 mg intravenously, followed by 100 mg infused over 60 min q12h; the daily dosage should not exceed 400 mg.

It is active against some strains of *Acinetobacter* spp. and *Stenotrophomonas* spp. and some Enterobacteriaceae. Minocycline yields higher blood levels than tigecycline and exhibits good lung penetration, measured as the epithelial lining fluid to serum ratio, thus making it an interesting therapeutic option for MDR *A. baumannii* VABP [82,83].

Minocycline has a better safety profile compared to polymyxins and aminoglycosides, as well as a higher bioavailability and better pharmacokinetics than tigecycline. Indeed, the latter has a strong activity against *A. baumannii* and *K. pneumoniae* but can achieve a serum concentration of only about 1.0 mg/L and does not undergo extensive renal elimination, limiting its use in treatment of BSI and cUTI. In contrast, a single 100 mg IV dose of minocycline can achieve serum concentration as high as 8.75 mg/L, and up to 400 mg daily can be administered [83]. Considering the scarcity of treatment options against *A. baumannii*, minocycline can play a role as a partner or as an alternative to colistin in novel combination therapy regimens, since it may not have consistent activity as monotherapy [84]. Minocycline IV formulation is not widely available, however.

Although primarily bacteriostatic, minocycline has synergistic bactericidal effects against MDR and XDR *A. baumannii* isolates when combined with colistin, carbapenems, and rifampicin and may be useful in treating severe *A. baumannii* infections, considering its high tissue penetration compared to other tetracyclines and the ability to continue oral administration after intravenous [85]. Moreover, when used in combination with other antimicrobials, minocycline can reduce the MIC value of companion agents, thus possibly attenuating the emergence of new drug resistance.

As of yet, the use of IV minocycline in patients with *A. baumannii* infections has not been examined in RCT. However, some reviews and retrospective studies have investigated its efficacy in this setting and have shown that IV minocycline was associated with high rates of clinical success and was well tolerated among patients with MDR *A. baumannii* infections. Ideally, clinical trials are needed to fully establish the real effectiveness and the place of IV minocycline in the treatment of these patients [86].

The most important feature of minocycline is represented by its good and well-documented safety profile, with an overall adverse event incidence rate of 13 per million per year. However, its use has been associated with hepatotoxicity, photosensitivity, skin discoloration, and drug-induced systemic lupus erythematosus [87].

### 4.7. Aztreonam

Aztreonam is the first member of a class of β-lactam antibiotics, the monobactams, developed several decades ago. By binding to PBP3, aztreonam inhibits the third and last stage of bacterial cell wall synthesis. Aztreonam is a drug of completely synthetic origin, and it has strong activity against susceptible gram Gram-negative bacteria, including non-XDR strains of *P. aeruginosa*. It is resistant to MBL but is inactivated by ESBL [63,88]. Because of this, it has been almost abandoned in the last three decades, when potent carbapenems have become available. However, aztreonam has recently regained attention due to the spread of MBL-producing carbapenem-resistant Gram-negatives. 

Aztreonam is marketed in the form of pharmaceutical formulations suitable for administration by both parenteral route or inhalation (indicated for treatment of respiratory symptoms in cystic fibrosis with *P. aeruginosa*) [89]. Therapeutic trials have shown aztreonam to be effective in Gram-negative infections including cUTI, in lower respiratory tract infections and in gynecological and obstetric, IAI, joint and bone, ABSSSI, uncomplicated gonorrhea and BSI.

In adults, the dose of aztreonam ranges from 500 mg to 2 g of the drug, intravenously, given every 6, 8, or 12 h. In patients with liver and/or kidney disease, dose reductions should be performed: for an eGFR < 30 mL/min, 2 g loading dose is followed by 1 g every 6–8 h; for an eGFR < 10 mL/min, a 2 g loading dose is followed by 0.5 g every 6–8 h [90].

Adverse events related to aztreonam administration have included *C. difficile* infection (pseudomembranous colitis), eosinophilia, skin rashes, and allergic reactions including anaphylaxis. Aztreonam therapy may also cause increased serum transaminases and alkaline phosphatase concentrations, hepatitis, and jaundice. Its activity against Gram-negative organisms is in general equal or superior to that observed with third-generation cephalosporins, cefotaxime, and ceftazidime [90].

### 4.8. Aztreonam-Avibactam

A real potential breakthrough in the treatment of XDR Gram-negative bacilli could be represented by the recent development of a new antimicrobial, made from the combination of the monobactam aztreonam with the non-β-lactam β-lactamase inhibitor avibactam. Aztreonam-avibactam is currently in clinical development for the treatment of serious infections caused by MBL-producing Enterobacterales, the most difficult-to-treat subtype of carbapenem-resistant bacilli for which therapeutic options are currently very limited [91]. A study by Karlowsky et al. tested for in vitro susceptibility to aztreonam-avibactam and comparator antimicrobial agents clinically significant isolates of Enterobacteriaceae (more than 50,000) and *P. aeruginosa* (more than 10,000) collected from hospitalized patients in hundreds of hospital laboratories worldwide between 2012 and 2015. About 99.8% of meropenem-nonsusceptible isolates (n = 1498) were inhibited by aztreonam-avibactam at a concentration of ≤8 μg/mL. Among these strains, there were >250 Enterobacterales isolates that were show by PCR and DNA sequencing to be positive for MBL genes (NDM, VIM, and IMP) [92]. This study demonstrates that aztreonam-avibactam possesses potent in vitro activity against a current global collection of Enterobacterales isolates, including meropenem nonsusceptible ones and MBL-positive isolates [93].

The rationale for this combination relies in the fact that aztreonam is stable to hydrolysis by MBLs, whilst avibactam effectively inhibits most other serine carbapenemases co-expressed with MBLs. Few clinical data are as of yet available regarding aztreonam-avibactam efficacy and safety, and results of ongoing studies are eagerly awaited.

### 4.9. Cefiderocol

Cefiderocol is a ‘siderophore’ cephalosporin. As other β-lactam antibiotics, cefiderocol exerts its bactericidal activity thorough inhibition of Gram-negative bacterial cell wall synthesis by binding to penicillin binding proteins. However, cefiderocol is unique in that it enters the bacterial periplasmic space after binding to extracellular iron and exploiting siderophore-like properties. This, coupled with enhanced stability to most β-lactamases, confer this drug a very high genetic barrier towards resistance [50]. Showing a chemical structure similar to both ceftazidime and cefepime, cefiderocol is resistant to hydrolysis by a variety of β-lactamases, including AmpC, ESBLs, and carbapenemases belonging to Ambler class A, B, and D. The resulting microbiological activity of cefiderocol includes most aerobic Gram-negative bacilli and is equal to or higher than that of ceftazidime-avibactam and meropenem. Specifically, at variance with both ceftazidime-avibactam and meropenem, cefiderocol is also effective in vitro against XDR *A. baumannii*. Cefiderocol’s activity against meropenem-nonsusceptible and *K. pneumoniae* carbapenemase (KPC)-producing Enterobacterales is comparable or superior to ceftazidime-avibactam. Cefiderocol also appears to be more potent in vitro than both ceftazidime-avibactam and meropenem against all resistance phenotypes of *P. aeruginosa* and against *S. maltophilia*.

The dosing regimen currently being used in phase III studies is 2 g administered intravenously every 8 h using a 3 h infusion. The mean plasma half-life (t½) is about 2.3 h, its protein binding is 60%, and its total drug clearance ranges from 4.6–6.0 L/h for both single- and multiple-dose infusions. Cefiderocol is primarily excreted unchanged by the kidneys (61–71%), similar to other β-lactams, with drug clearance decreasing with impaired creatinine clearance. Dose adjustment is thus required for patients with moderate to severe renal impairment.

Cefiderocol has performed similarly to or has been superior to comparator agents, including ceftazidime and cefepime. Cefiderocol appears to be well tolerated (reported adverse effects included gastrointestinal symptoms and phlebitis), with a side-effect profile comparable to other cephalosporins [94].

Despite of these promising early clinical and PK/PD data, a recent clinical trial did not support the higher effectiveness of cefiderocol in severe infections due to XDR bacteria in critically ill subjects [95]. Further clinical data are needed to better understand the role of this novel option in the XDR infection treatment scenario.

### 4.10. Eravacycline

Eravacycline is a newly developed, fully synthetic tetracycline derivative, showing potent broad-spectrum activity against a wide variety of microorganisms, including ESBL-producing Enterobacterales and *A. baumannii*. Eravacycline also has activity against many Gram-positive organisms, such as MRSA and VRE, and anaerobes such as *Bacteroides* spp. [91,96].

Recently approved for use in cIAIs and cUTI, eravacycline has been compared to ertapenem and meropenem for the former and to levofloxacin for the latter. Eravacycline appeared to be noninferior to ertapenem but did not meet noninferiority criteria when compared to levofloxacin for cUTI.

This novel tetracycline has a half-life of 20 h and a protein binding of about 80% [97]. The recommended dosage regimen is thus 1 mg/kg every 12 h by intravenous infusion over 1 h. In clinical studies, eravacyclin has been administered for a duration of 4–14 days. Interestingly, it does not require dose adjustments based on renal function.

Eravacycline has demonstrated an acceptable tolerability profile, with infusion site reactions, nausea, vomiting, and diarrhea being the most common adverse reactions. Most of these have actually been of mild to moderate severity, in line with the experience with other tetracyclines. The favorable profile of this novel molecule also stems from its broad spectrum of activity against several clinically relevant pathogens, including those expressing some of the tetracycline-specific resistance mechanisms. In addition, eravacycline appeared to be more potent in vitro than tigecycline [98]. Whether comparative clinical data will show superiority and better safety of eravacycline over tigecyline for the early empirical treatment of cIAIs, especially nosocomially-acquired cases, remains to be determined [99].

## 5. Conclusions

An exciting scenario is opening today with the long-awaited growing availability of novel molecules for the treatment of AMR bacteria. Knowledge of mechanisms of action and resistance patterns will allow physicians to increasingly drive antimicrobial treatment towards a precision medicine approach. Strict adherence to antimicrobial stewardship practices will allow us to preserve the emerging antimicrobials for our future.

Undoubtedly, and as recently pointed out by an ad hoc World Health Organization panel, very few of the antibiotics currently in clinical development show an innovative mechanism of action [100]. Most represent an evolution of prior molecules against which antimicrobial resistance is already emerged. Therefore, in order to successfully address the problem of drug resistance, it is of outmost importance to develop truly innovative molecules. At present, the most powerful strategy in our hands remains the correct and judicious use of available antibiotics. How long it is going to take for MDR bacteria to develop resistance even to newer molecules remains to be determined [5].

## Figures and Tables

**Table 1:** Common MDR/XDR microorganisms of clinical relevance, main resistance mechanisms, and current/emerging antimicrobial options.

	Mechanism(s) of Resistance	Current Options	Emerging Options
*GRAM-POSITIVE COCCI*			
Methicillin-resistant staphylococci (MRSA, MRCoNS)	PBP2a expression	Daptomycin, Linezolid	Ceftaroline, Ceftobiprole, Tedizolid, Dalbavancin, Fosfomycin
Vancomycin intermediate *Staphylococcus aureus* (VISA)	Chromosomal mutations	Daptomycin, Linezolid	Tedizolid, Dalbavancin
Vancomycin resistant *Staphylococcus aureus* (VRSA)	*vanA* gene expression	Daptomycin, Linezolid	Tedizolid, Dalbavancin
Ampicillin-resistant enterococci (ARE)	PBP mutation/overexpression	Vancomycin, Linezolid, Daptomycin	Dalbavancin, Ceftobiprole
Vancomycin-resistant enterococci (VRE)	*vanA*, *vanB* gene expression	Linezolid, Daptomycin	Tedizolid, Dalbavancin
Penicillin-resistant *Streptococcus pneumoniae* (PRSP)	PBP mutation	Ceftriaxone	Ceftaroline, Ceftobiprole, Tedizolid
*GRAM-NEGATIVE BACILLI*			
Carbapenem-resistant Enterobacterales	β-lactamase production	Colistin, Tigecycline	KPC/OXA-48: Ceftazidime-Avibactam KPC: Meropenem-Vaborbactam KPC: Imipenem-Relebactam MBL: Aztreonam-Avibactam Fosfomycin Eravacycline Plazomicin Cefiderocol
XDR *Pseudomonas aeruginosa*	β-lactamase production Porin loss/mutation Efflux pump expression	Colistin	Ceftolozane-Tazobactam Ceftazidime-Avibactam Aztreonam/Aztreonam-Avibactam Fosfomycin Cefiderocol
*Acinetobacter baumannii*	β-lactamase production Porin loss/mutation Efflux pump expression	Colistin, Tigecycline	Cefiderocol Minocycline Eravacycline
*Stenotrophomonas malthophilia*/*Burkolderia cepacia*	β-lactamase production Porin loss/mutation Efflux pump expression	Co-trimoxazole	Cefiderocol Ceftazidime-Avibactam
